# Supplementary material for: The Equine Faecal Microbiota of Healthy Horses and Ponies in The Netherlands: Impact of Host and Environmental Factors
Source: Animals (Basel). 2021 Jun 12;11(6):1762. doi: 10.3390/ani11061762 (PMC8231505; doi:10.3390/ani11061762)
Supplement: Supplementary file 1 [file animals-11-01762-s001.zip › animals-1242810-supplementary.pdf]

Supplementary Figure S1 Relative abundance of bacterial communities at (**A**) phylum- and (**B**) class level in the microbiota of fecal samples collected from ponies at different time points after exposure to air. The phylum of Bacteroidetes decreased in relative abundance at t=12h and t=24h compared to t=0, while Firmicutes increased. At class level Bacilli increased after 12h of air exposure.

(**A**)

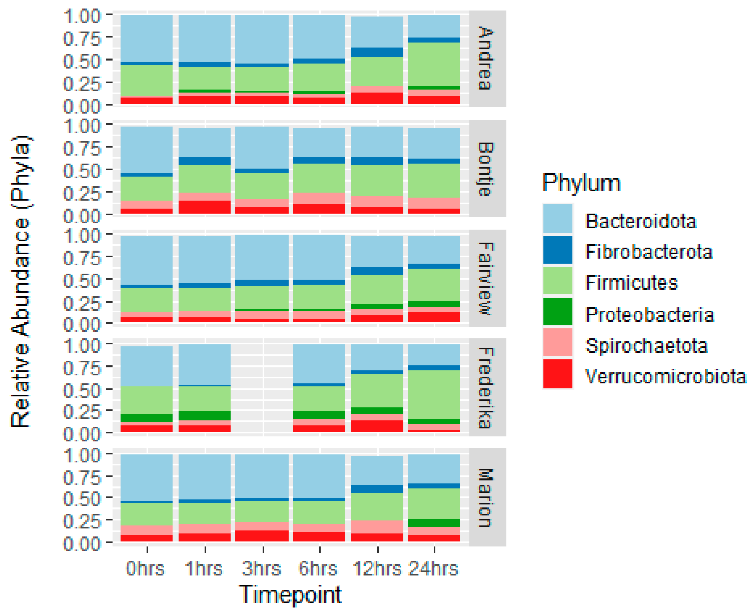

(**B**)

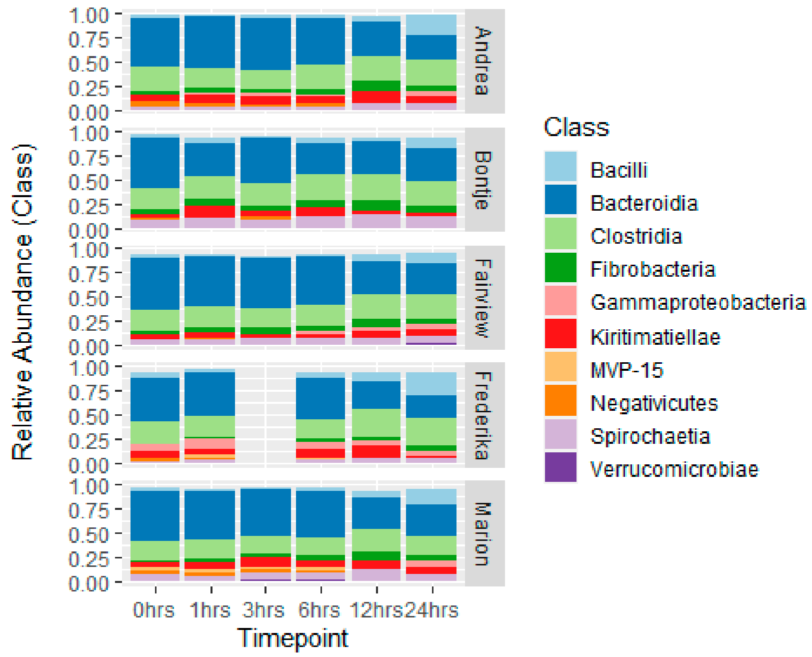

Supplementary Figure S2. Relative abundance of most abundant phyla and classes in the fecal microbiota of 79 horses and ponies in the Netherlands. (**A**) 61 equids at farm I and 18 equids at farm II - phylum level (**B**) 61 equids at farm II and 18 equids at farm II – class level

(**A**)

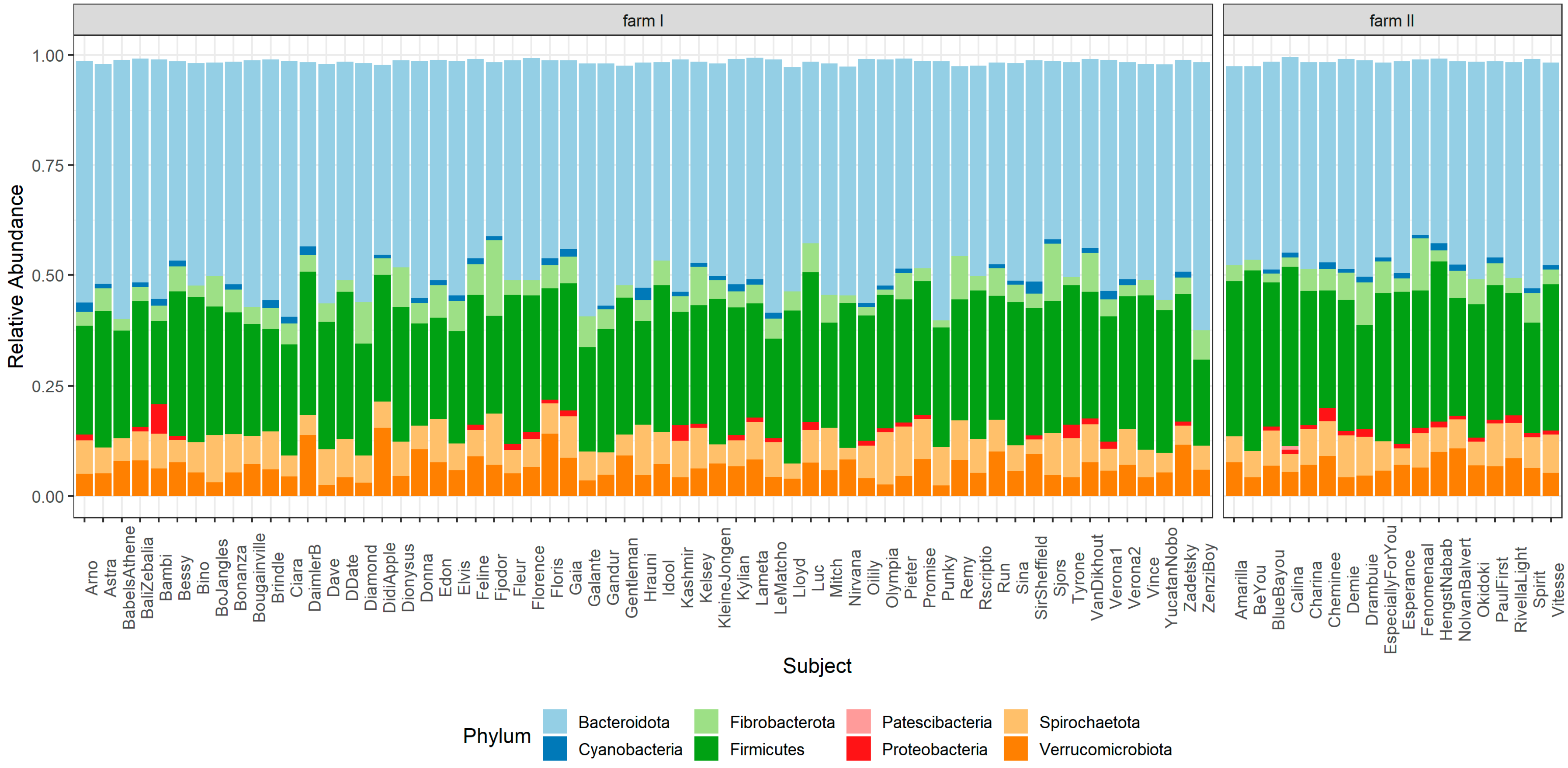

(B)

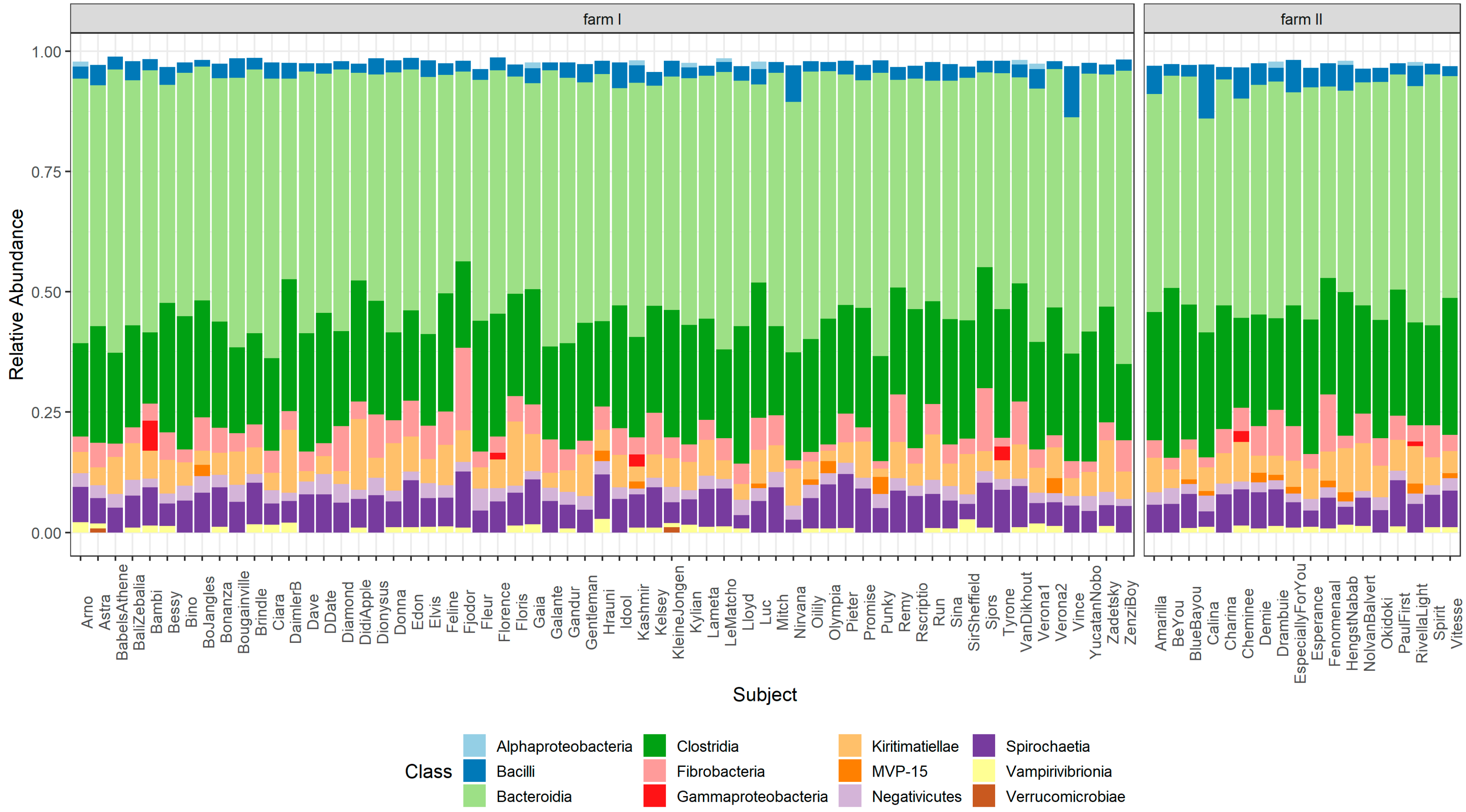

farm I
farm II
Relative Abundance
1.00
0.75
0.50
0.25
0.00
Arno
Astra
BabelsAthene
BaliZebalia
Bambi
Bessy
Bino
BoJangles
Bonanza
Bougainville
Brindle
Ciara
DaimlerB
Dave
DDate
Diamond
DidiApple
Dionysus
Donna
Edon
Elvis
Feline
Fjodor
Fleur
Florence
Floris
Gaia
Galante
Gandur
Gentleman
Hrauni
Idool
Kashmir
Kelsey
KleineJongen
Kylian
Lameta
LeMatcho
Lloyd
Luc
Mitch
Nirvana
Oilily
Olympia
Pieter
Promise
Punky
Remy
Rscriptio
Run
Sina
SirSheffield
Sjors
Tyrone
VanDikhout
Verona1
Verona2
Vince
YucatanNobo
Zadetsky
ZenziBoy
Amarilla
BeYou
BlueBayou
Calina
Charina
Cheminee
Demie
Drambuie
EspeciallyForYou
Esperance
Fenomenaal
HengstNabab
NolvanBalvert
Okidoki
PaulFirst
RivellaLight
Spirit
Vitesse
Subject
Class
Alphaproteobacteria
Bacilli
Bacteroidia
Clostridia
Fibrobacteria
Gammaproteobacteria
Kiritimatiellae
MVP-15
Negativicutes
Spirochaetia
Vampirivibrionia
Verrucomicrobiae
